# Risk factors and surgical outcomes in periocular basal cell carcinoma treated with Mohs micrographic surgery

**DOI:** 10.1016/j.abd.2025.501281

**Published:** 2026-01-27

**Authors:** Glaysson Tassara Tavares, Alberto Julius Alves Wainstein, João Renato Vianna Gontijo, Ana Paula Drumond Lage

**Affiliations:** a Postgraduate Institute, Faculdade Ciências Médicas de Minas Gerais, Belo Horizonte, MG, Brazil; b Dermatology Service, Hospital das Clínicas, Universidade Federal de Minas Gerais, Belo Horizonte, MG, Brazil

Dear Editor,

Periocular basal cell carcinoma (BCC) represents a therapeutic challenge due to its invasive behavior and proximity to critical anatomical structures. Few studies have evaluated its predictors of aggressiveness and recurrence treated with Mohs micrographic surgery (MMS).

A retrospective cohort study was conducted at a Brazilian multidisciplinary center by a single surgeon (maintaining the same surgical technique standard). Patients with periocular BCC treated with MMS between 2008 and 2018 were included.

Data were collected on sex, age, tumor location, aggressive histological subtype (micronodular, sclerosing, infiltrative, and metatypical/basosquamous) or non-aggressive (superficial, nodular), tumor diameter (> 10 mm or ≤ 10 mm), prior Mohs surgery recurrence (PMSR), MMS stages, type of reconstruction, follow-up, and postoperative recurrence. Cases with perineural invasion (two) and prior radiotherapy (none) were excluded because they are already established high-risk factors, for homogenization of the statistical sample, and because there were few cases. In cases with mixed subtypes, the most aggressive was considered. Reconstructions were classified based on non-standardized criteria as minor (primary closure, small flaps, canthotomy-cantholysis), moderate (Tenzel flap), and significant (Hughes or Mustardé flaps), according to the size of the defect and structures involved (up to 25%–30%, 30% to 60%, and above 60% of the eyelid margin, respectively). For cases without involvement of the eyelid margin, those requiring the Mustardé flap were considered significant, while the others were considered minor.

The statistical analysis included descriptive statistics, the Shapiro-Wilk test, the Wilcoxon test, Fisher's exact test, and the Kruskal-Wallis test (p < 0.05). Logistic regression models were used, with variable selection based on the calculation of Akaike's information criterion.

One hundred and eight patients were included, 57.1% female, mean age 66.3 years (±15.1; 27–91 years). The lower eyelid was the most affected site (55.5%) and medial canthus (29.7%), consistent with other literature studies on MMS in this location (51%).^1^ Aggressive subtypes were present in 66.1%. Tumors with a diameter > 10 mm were observed in 49.1%, and 8% presented with PMSR. Recurrence after MMS occurred in 3.8% (n = 4), with a follow-up of 64.8 months (±31.6), similar to previous studies with recurrence of 3%–3.3% in similar cohorts.^2^, ^3^ The mean tumor area was 96 mm^2^ and the mean final area was 398 mm^2^. Reconstructions were minor (62%), moderate (13%), and significant (25%).

More stages of MMS were associated with PMSR (2.9 ± 1.5) vs. primary BCC (2.0 ± 1.1; p = 0.002), diameter > 10 mm (2.6 ± 1.4) vs. < 10 mm (1.9 ± 1.1; p = 0.007), and aggressive histology (2.4 ± 1.1) vs. non-aggressive (2.0 ± 1.5; p = 0.005).

Table 1, through univariate logistic regression, exemplifies that tumors > 10 mm were strongly associated with aggressive histology (OR = 7.33; p < 0.001) and larger defect area (OR = 1.008; p < 0.001). Tumors with PMSR showed a greater number of stages (OR = 1.72; p = 0.002), a larger defect area (OR = 1.008; p = 0.001), and more significant reconstructions (OR = 2.73; p = 0.039). Tumors with aggressive histology exhibited a greater chance of complex reconstructions (OR = 4.53; p = 0.006).

**Table 1:** Univariate logistic regression model of variables associated with the main surgical outcomes in periocular basal cell carcinoma. Pre-operative Mohs surgical recurrence (PMSR), Odds ratio (OR).

Outcome	Variable	p-value	Odds ratio (OR)	Confidence Interval
Diameter > 10 mm	PMSR	<0.001	5.875	2.413-15.635
	Stages	0.008	1.575	1.142-2.256
	Defect area	<0.001	1.008	1.005-1.012
	Aggressive histology	<0.001	7.333	3.093-18.848
	Moderate reconstruction	0.829	0.876	0.246-2.832
	Significant reconstruction	<0.001	6.938	2.494-22.807
PMSR	Diameter >10 mm	<0.001	5.875	2.413-15.635
	Stages	0.002	1.721	1.236-2.485
	Defect area	0.001	1.008	1.005-1.012
	Aggressive histology	0.158	1.873	0.8-4.623
	Moderate reconstruction	0.154	2.391	0.697-7.945
	Significant reconstruction	0.039	2.732	1.05-7.169
Aggressive histology	Diameter > 10 mm	<0.001	7.333	3.093-18.848
	Stages	0.072	1.364	0.988-1.954
	Defect area	0.066	1.001	1-1.002
	PMSR	0.158	1.873	0.8-4.623
	Moderate reconstruction	0.056	3.778	1.067-17.817
	Significant reconstruction	0.06	4.533	1.636-14.815

Table 2 using the multivariate model indicated that PMSR was associated with a larger defect area (OR = 1.002; p = 0.001) and significant reconstruction (OR = 3.77; p = 0.0177), as well as previous studies that demonstrated that lesions previously inadequately treated by conventional surgery tend to exhibit increased/more aggressive subclinical growth and behavior when recurring locally.^4^, ^5^ Tumors > 10 mm were associated with PMSR (OR = 8.02; p = 0.0148), aggressive subtype (OR = 8.41; p = 0.0021) and larger defect area (OR = 1.006; p = 0.0017). Aggressive histology was associated with a diameter > 10 mm (OR = 6.92; p < 0.001).

**Table 2:** Multivariate logistic regression for outcomes of diameter > 10 mm, prior Mohs micrographic surgery recurrence (PMSR), and aggressive histological subtype. Odds ratio (OR).

Outcome	Variable	Odds Ratio (OR)	Confidence Interval	p-value
Diameter > 10 mm	Defect area	1.006	1.003–1.010	0.0017
	PMSR	8.023	1.688–52.422	0.0148
	Aggressive histology	8.412	2.316–36.826	0.0021
PMSR	Defect area	1.002	1.001–1.004	0.0007
	Significant reconstruction	3.765	1.256–11.510	0.0177
Aggressive histology	Diameter > 10 mm	6.922	2.724–19.070	<0.001
	Moderate reconstruction	4.944	1.271–24.944	0.0305

The anatomical distribution and cure rate (3.8%) after MMS are aligned with the literature, as in Shi et al. 4.2% (n = 167), with the lower eyelid and medial canthus being the most frequent sites.^1^, ^2^, ^3^, ^6^, ^7^, ^8^ The initial descriptive analysis revealed associations between a higher number of MMS stages and tumors with aggressive histology, diameter > 10 mm, and PMSR, as described in previous studies.^4^, ^9^

The univariate analysis reinforced that larger tumors, aggressive histology, or those with PMSR exhibit a greater chance of subclinical growth and complex reconstructions.

The multivariate analysis confirmed these findings; however, it showed that tumors with PMSR and tumors > 10 mm were almost seven times more likely to have aggressive histology. Although modest, the differences in the area of ​​the final defect were statistically significant, highlighting the impact of these tumor characteristics on surgical extent. The findings suggest that larger tumors with aggressive histology require wider excisions.

Significant reconstructions were associated with PMSR (OR = 3.76), suggesting that recurrent tumors require more complex approaches. However, reconstructive complexity may reflect tumor biology/behavior (size, histological subtype), without being an independent predictor. As shown above, BCCs with PMSR tend to be aggressive subtypes and have a diameter > 10 mm.

The association between size, PMSR, and tumor aggressiveness reaffirms MMS as the preferred technique in these cases, given the higher chance of subclinical growth.^4^, ^5^, ^9^ Margin control is crucial, especially where conventional surgery is still common and access to MMS is limited, reducing the risks of invasive tumors.^2^, ^3^

The study limitations include its retrospective and single-center design, which may limit its findings. Larger studies may validate these associations and support individualized surgical planning.

## ORCID ID

Alberto Julius Alves Wainstein: 0000-0002-8227-7972

Ana Paula Drumond Lage: 0000-0002-0389-2433

Joao Renato Vianna Gontijo: 0000-0001-5544-7107

Glaysson Tassara Tavares; 0000-0002-1688-2955

## Financial support

None declared.

## Authors' contributions

Glaysson Tassara Tavares: Drafting and editing of the manuscript; critical review of important intellectual content and approval of the final version of the manuscript.

Alberto Julius Alves Wainstein: Drafting and editing of the manuscript; critical review of important intellectual content and approval of the final version of the manuscript.

João Renato Vianna Gontijo: Drafting and editing of the manuscript; critical review of important intellectual content and approval of the final version of the manuscript.

Ana Paula Drumond Lage: Drafting and editing of the manuscript; critical review of important intellectual content and approval of the final version of the manuscript.

## Research data availability

The entire dataset supporting the results of this study was published in this article.

## Conflicts of interest

None declared.
